# Supplementary material for: SpookyNet: Learning force fields with electronic degrees of freedom and nonlocal effects
Source: Nat Commun. 2021 Dec 14;12:7273. doi: 10.1038/s41467-021-27504-0 (PMC8671403; doi:10.1038/s41467-021-27504-0)
Supplement: Supplementary file 1 — Supplementary Information [file 41467_2021_27504_MOESM1_ESM.pdf]

Supplementary Information

SpookyNet: Learning Force Fields with Electronic Degrees of Freedom and Nonlocal Effects

Oliver T. Unke *et al.*

SUPPLEMENTARY DISCUSSION

Completeness of atomic descriptors in SpookyNet

Many ML algorithms for constructing potential energy surfaces make use of some sort of descriptor to represent atoms in their chemical environment. As long as this description is *complete*, any atom-centered property (including atomic decompositions of extensive properties such as energy) can be predicted from the descriptors.[2] In this context, *completeness* means that structures which are not convertible into each other (by translations, rotations, or permutations of equivalent atoms) map to different descriptors.

A simple descriptor for the environment of an atom i at position $\vec{r}_i$ consists of the set of distances $r_{ij} = \|\vec{r}_{ij}\|$ (with $\vec{r}_{ij} = \vec{r}_j - \vec{r}_i$) to neighboring atoms j, and the set of angles

$$\alpha_{ijk} = \arccos\left(\frac{\langle \vec{r}_{ij}, \vec{r}_{ik}\rangle}{\|\vec{r}_{ij}\|\|\vec{r}_{ik}\|}\right) \tag{1}$$

between all possible combinations of neighboring atoms j and k. For environments consisting of multiple different species, separate sets of distances (and angles) are necessary for each element (or combination of elements). However, for simplicity, it is assumed here that all atoms are identical. A disadvantage of using angles in the descriptor is that their computation scales $\mathcal{O}(n^2)$ with the number of neighbors n, because all combinations must be considered. An alternative to encode angular information, which scales $\mathcal{O}(n)$, is to replace the set of angles with invariants of the form

$$a_{i,l} = \sum_{m=-l}^{l}\left(\sum_{j=1}^{n} Y_l^m(\vec{r}_{ij})\right)^2 \tag{2}$$

derived from the angular power spectrum, where Y_l^m are the spherical harmonics (see Eq. 17 in the main text). In the following, $a_{i,l}$ for $l = 0, 1, 2, 3, 4$ are called s, p, d, f, and g invariants because of their relation to the symmetries of atomic orbitals. The disadvantage here is that when using a finite number ($l = 0, \ldots, L$) of power spectrum invariants as angular descriptor, some environments with different sets of angles may lead to the same descriptor. For example, square planar and terahedral environments have the same s and p invariants ($L = 1$), so it is necessary to include at least d invariants ($L = 2$) in the descriptor to differentiate them (see Fig. 1B).

There is a widespread belief in the literature that sets of distances and angles are sufficient for a *complete* description of atomic environments.[3,4] However, it was recently demonstrated that this is not the case, and even including the set of dihedrals

$$\delta_{ijkl} = \arccos\left(\frac{\langle \vec{r}_{ij} \times \vec{r}_{ik}, \vec{r}_{ik} \times \vec{r}_{il}\rangle}{\|\vec{r}_{ij} \times \vec{r}_{ik}\|\|\vec{r}_{ik} \times \vec{r}_{il}\|}\right) \tag{3}$$

between triplets of neighboring atoms does not lead to a *complete* description in general.[1] In this context, it is interesting to investigate the *completeness* of the atomic descriptors $\mathbf{f}$ (see Eq. 3 in the main text) learned by SpookyNet and compare its ability to distinguish different structures to other popular approaches. For this purpose, five pairs of distinct atomic environments (shown in Fig. 1), with geometries that are particularly difficult to separate, are considered. Then, different models are trained to predict scalar labels of 1 (for one of the environments) and −1 (for the other environment) from the descriptors of the central atoms (blue) in each pair. It can be observed that models either learn to predict the labels with virtually zero error (up to numerical precision), i.e. the environments can be distinguished, or a value of 0 is predicted for both central atoms, i.e. their environments are mapped to the same descriptor and a compromise between the contradictory labels has to be found. The results are summarized in Table 1. For evaluating PhysNet[5] and DimeNet,[6] the reference implementations available from `https://github.com/MMunibas/PhysNet` and `https://github.com/klicperajo/dimenet` are used. PaiNN[7] and NequIP[8] are evaluated using in-house implementations. For BPNN and SchNet, the implementations available in SchNetPack[9] are used. The FCHL18/19[10,11] models are evaluated using the QML package.[12] All models were trained on multiple randomly rotated versions of the environments shown in Fig. 1. This was done to prevent models picking up on differences due to floating point imprecision, which otherwise may make environments distinguishable even when their descriptors are degenerate (up to numerical noise).

Most models based on hand-crafted descriptors can only distinguish environments when their sets of distances and angles (and in some cases dihedrals) differs. Message-passing neural networks (MPNNs) on the other hand can learn to distinguish all environments shown in Fig. 1, provided that at least $T \geq 2$ message-passing steps are used. The amount of information that can be resolved with a single message-passing step ($T = 1$) is often related to the power spectrum invariants (see Eq. 2) and different MPNNs mainly differ in the maximum order L which they can resolve in a single update (with the exception of DimeNet,[6] which uses angles directly but scales $\mathcal{O}(n^2)$ with the number of neighbors n). SpookyNet uses an update with a maximum order of $L = 2$, which is sufficient to differentiate most common chemical environments (as long as they are distinguishable by distances and angles). It would be possible to introduce higher order interactions with the symmetry of f-, or even g-orbitals into the update step (see Eq. 12 in the main text), so that additional environments (e.g. Fig. 1d) become distinguishable with a single update, but this increases the computational cost and is found to give little benefit (in terms of additional accuracy for predictions) in practice.

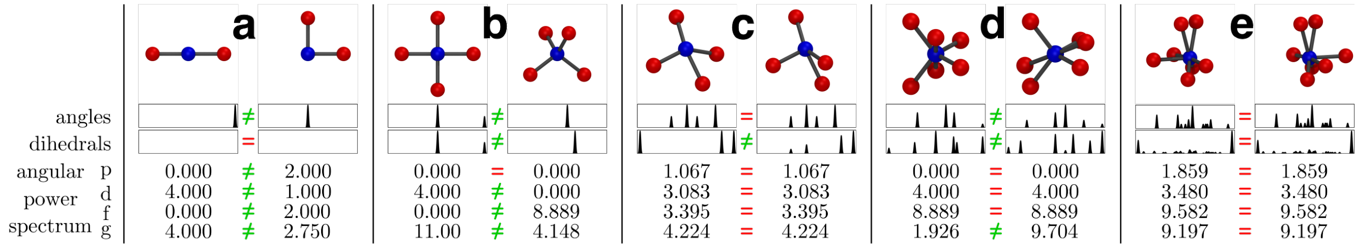

SUPPLEMENTARY FIG. 1: Pairs of distinct atomic environments where all neighboring atoms (red) have the same distance from the central atom (blue). The distributions of angles (Eq. 1) and dihedrals (Eq. 3) are visualized and values of the angular power spectrum invariants (Eq. 2) for different angular momenta $l = 1, \dots 4$ (p, d, f, g) are given for each structure (the s invariant simply counts the number of neighbors and is therefore omitted). Since all distances to neighboring atoms are identical, descriptors need to be able to at least resolve angular information to distinguish the structures (**a**). However, for some structures, the power spectrum invariants may be degenerate for small values of l (**b** and **d**). Some structures even have identical angular distributions, in which case the power spectrum invariants are equal for all $l = 0, \dots, \infty$ and information about dihedrals is necessary to distinguish the environments (**c**). Note that some environments cannot even be distinguished when information about dihedrals is included (**e**).[1]

**model**	**scaling**	**A**	**B**	**C**	**D**	**E**
**hand-crafted descriptors**						
BPNN[13]	$\mathcal{O}(n^2)$	✓	✓	✗	✓	✗
FCHL19[11]	$\mathcal{O}(n^2)$	✓	✓	✗	✓	✗
FCHL18[10]	$\mathcal{O}(n^3)^*$	✓	✓	✓**	✓	✗
**learned descriptors**						
SchNet[14]	$\mathcal{O}(n)$	(✓)	(✓)	(✓)	(✓)	(✓)
PhysNet[5]	$\mathcal{O}(n)$	(✓)	(✓)	(✓)	(✓)	(✓)
DimeNet[6]	$\mathcal{O}(n^2)$	✓	✓	(✓)	✓	(✓)
NequIP[8]	$\mathcal{O}(n)$	✓	(✓)	(✓)	(✓)	(✓)
PaiNN[7]	$\mathcal{O}(n)$	✓	(✓)	(✓)	(✓)	(✓)
SpookyNet	$\mathcal{O}(n)$	✓	✓	(✓)	(✓)	(✓)

* when dihedrals are included
** only distinguishable with dihedrals

SUPPLEMENTARY TABLE 1: Ability of models to differentiate the atomic environments shown in Fig. 1 (✓: distinguishable, ✗: indistinguishable) and the scaling of their computational cost with respect to the number of neighbors n. Message-passing neural networks with learned descriptors can distinguish all environments when there are $T \geq 2$ message-passing steps. However, when only a single step is used ($T = 1$), the environments marked with (✓) become indistinguishable.

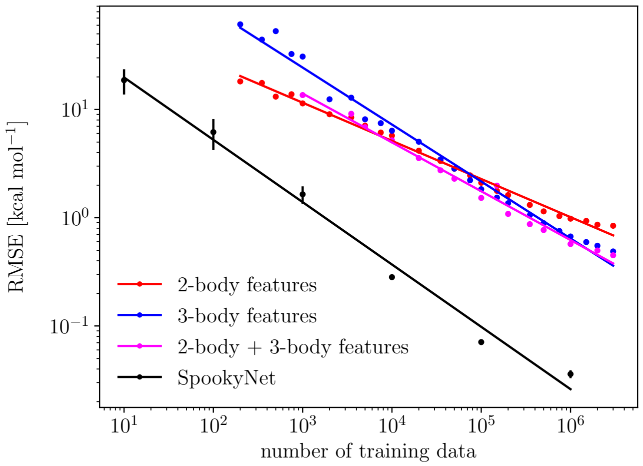

SUPPLEMENTARY FIG. 2: Energy learning curves for the random CH_4 dataset[15] suggested in Ref. 1. SpookyNet (black) is compared to feedforward neural networks trained on 2-body and/or 3-body features (red, blue, magenta), see Ref. 1 for details.

Pozdnyakov *et al.* propose a different *completeness* test based on a data set of ~7.7M CH_4 structures[15] that were generated by randomly placing H atoms in a 3 Å sphere around the C atom (see Ref. 1 for details). Due to the strongly distorted geometries, potential energies in this data set vary by ~1400 kcal mol^{-1} and forces by ~10700 kcal mol^{-1} Å^{-1}. Further, this way of sampling will lead to many structures with (nearly) degenerate sets of angles (see Fig. 1c) and is thus particularly challenging to learn. For this task, it is to be expected that models relying on *incomplete* descriptors improve at a slower rate (and eventually cease to improve at all) when increasing the number of training data.[1] The performance of SpookyNet on this data set for different training set sizes is summarized in Table 2. With only 10 training points (~0.00013% of the data), SpookyNet reaches prediction errors that correspond to a relative absolute error of just ~1% (with respect to the energy range covered in the data set). Chemical accuracy (absolute errors <1 kcal mol^{-1}) is reached with as few as 1000 training points. The learning curve (see Fig. 2) shows that the performance of SpookyNet increases steadily when more data is used for training while being about two orders of magnitude more data-efficient than other methods. The increased data efficiency is largely due to a much lower y-axis intercept, which indicates a high target similarity of the learned descriptor.[16]

n_{train}	**energy** [kcal mol^{-1}] MAE	RMSE	**forces** [kcal mol^{-1} Å^{-1}] MAE	RMSE
10	11.698 (2.440)	18.650 (4.889)	14.426 (3.280)	40.302 (16.473)
100	4.011 (1.688)	6.183 (1.969)	5.782 (1.436)	14.345 (4.730)
1 000	0.607 (0.030)	1.646 (0.304)	1.360 (0.039)	5.326 (2.614)
10 000	0.078 (0.002)	0.282 (0.009)	0.249 (0.007)	0.998 (0.032)
100 000	0.020 (0.001)	0.071 (0.003)	0.071 (0.001)	0.326 (0.012)
1 000 000	0.020 (0.002)	0.036 (0.003)	0.063 (0.006)	0.165 (0.015)

SUPPLEMENTARY TABLE 2: Mean absolute errors (MAEs) and root mean square errors (RMSEs) of energies and forces for the random CH_4 dataset[15] suggested in Ref. 1. Results are averaged over 16 ($n_{\text{train}} = 10$), 8 ($n_{\text{train}} = 100$), or 4 ($n_{\text{train}} \geq 1000$) random splits and the standard deviation between runs is given in brackets.

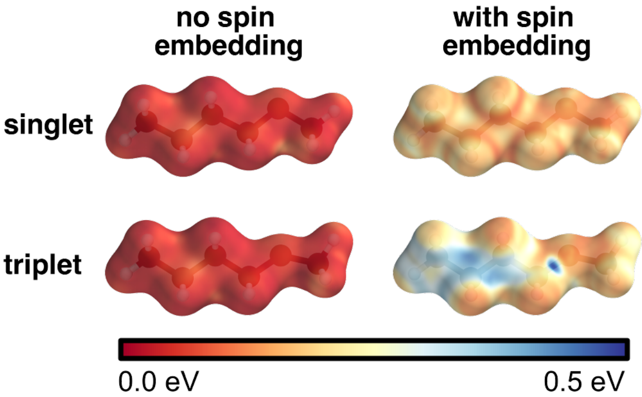

SUPPLEMENTARY FIG. 3: Local chemical potential for a random carbene chosen from the QMSpin database (the optimized geometries for the singlet/triplet states are shown). The chemical potential for a model without spin embeddings lacks features, whereas a model with spin embeddings learns a rich representation with significant differences between singlet and triplet states.

Conformer benchmark

As an additional test of extrapolation to larger molecules, the different models trained on QM7-X were applied to structures from the conformer benchmark introduced in Ref. 20. All structures that were non-neutral or contained elements other than H, C, N, O, S, or Cl were filtered out, because they are not covered by the QM7-X data set. In total, 5178 structures with an average number of 23 non-hydrogen atoms are considered (the largest structure contains 48 non-hydrogen atoms). All models were evaluated using the metrics introduced in Ref. 20 and compared to reference data computed at the same level of theory as QM7-X (see Ref. 19 for details). Both SpookyNet and PaiNN predict relative energies with sub-kcal accuracy (see Table 5), however, all models systematically overpredict absolute energies for systems larger than those contained in QM7-X (SpookyNet: 0.92 kcal/mol/atom, PaiNN: 1.12 kcal/mol/atom, SchNet: 3.78 kcal/mol/atom). As such, training on QM7-X is not sufficient if absolute energies of large structures are of interest.

In this context, it is also illuminating to compare wall clock times. All DFT reference calculations were performed on 72-core Intel Xeon IceLake-SP processors and took on average 24 min to complete. In contrast, evaluating a single structure with SpookyNet on a 6-Core Intel Core i7 takes 30 ms on average (speedup w.r.t. DFT $> 10^4$). However, it is usually more efficient to evaluate multiple structures in parallel on a GPU. For example, with a batch size of 250 structures, evaluating a single structure on an NVIDIA A100 SXM4 40 GB GPU takes only 0.25 ms on average (speedup w.r.t. DFT $> 10^6$).

Why the name SpookyNet?

In a famous letter to Max Born, Albert Einstein referred to the nonlocal nature of quantum systems as "*spooky actions at a distance*". SpookyNet also incorporates nonlocality in its architecture, for example by "spreading" (or rather delocalizing) electronic information over atoms and allowing nonlocal interactions between them, hence the name.

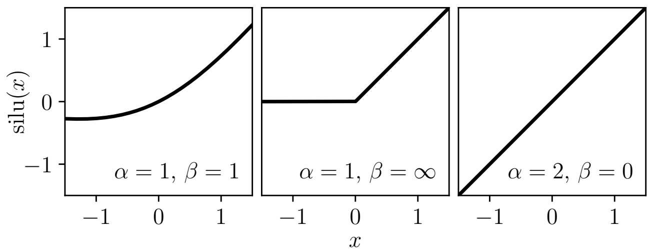

SUPPLEMENTARY FIG. 4: Generalized SiLU activation (Eq. 6 in the main text). For $\alpha = 1, \beta = \infty$, silu($x$) is equivalent to $\max(x, 0)$ (also known as ReLU activation), whereas for $\alpha = 2, \beta = 0$, the identity function is obtained.

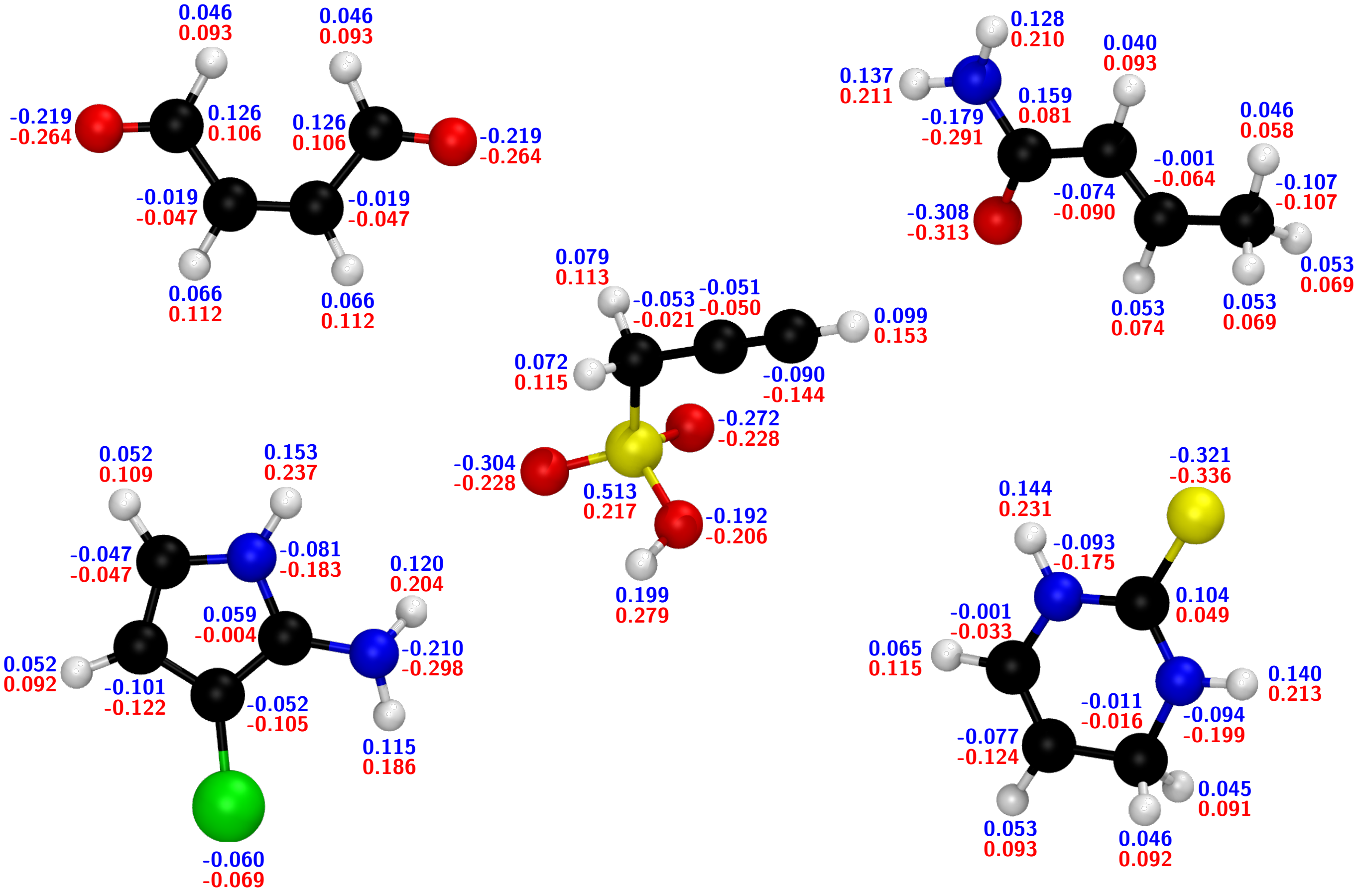

SUPPLEMENTARY FIG. 5: Comparison of partial charges predicted by SpookyNet (red) with Hirshfeld[17] charges (blue) for five molecules drawn from the QM7-X dataset.[18] Note that SpookyNet learns to perform the charge decomposition automatically by matching the molecular dipole moment and is not trained to reproduce Hirshfeld charges.
(H: white, C: black, N: blue, O: red, S: yellow, Cl: green)

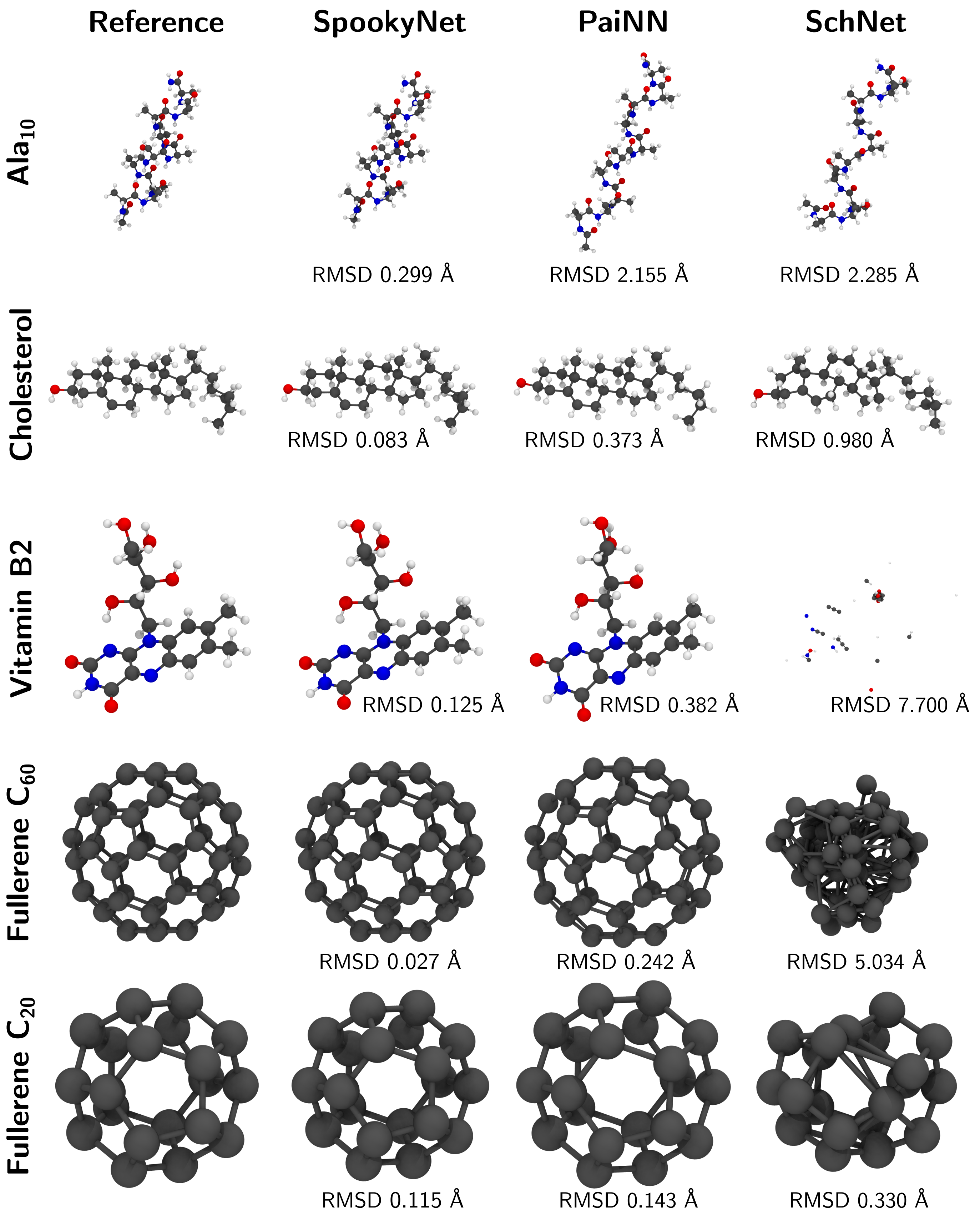

SUPPLEMENTARY FIG. 6: Optimized geometries and root mean square deviations (RMSD) for several molecules obtained from different models trained on the QM7-X dataset. The reference geometry was calculated at the same level of theory as QM7-X (PBE0-MBD).

element		Z	1s	2s	2p	3s	3p	4s	3d	4p	5s	4d	5p	6s	4f	5d	6p	vs	vp	vd	vf	
H	$\mathbf{d}'_1 =$ [	1	1	0	0	0	0	0	0	0	0	0	0	0	0	0	0	1	0	0	0	]$^\mathsf{T}$
C	$\mathbf{d}'_6 =$ [	6	2	2	2	0	0	0	0	0	0	0	0	0	0	0	0	2	2	0	0	]$^\mathsf{T}$
N	$\mathbf{d}'_7 =$ [	7	2	2	3	0	0	0	0	0	0	0	0	0	0	0	0	2	3	0	0	]$^\mathsf{T}$
O	$\mathbf{d}'_8 =$ [	8	2	2	4	0	0	0	0	0	0	0	0	0	0	0	0	2	4	0	0	]$^\mathsf{T}$
P	$\mathbf{d}'_{15} =$ [	15	2	2	6	2	3	0	0	0	0	0	0	0	0	0	0	2	3	0	0	]$^\mathsf{T}$
S	$\mathbf{d}'_{16} =$ [	16	2	2	6	2	4	0	0	0	0	0	0	0	0	0	0	2	4	0	0	]$^\mathsf{T}$
Fe	$\mathbf{d}'_{26} =$ [	26	2	2	6	2	6	2	6	0	0	0	0	0	0	0	0	2	0	6	0	]$^\mathsf{T}$
I	$\mathbf{d}'_{53} =$ [	53	2	2	6	2	6	2	10	6	2	10	5	0	0	0	0	2	5	10	0	]$^\mathsf{T}$
Au	$\mathbf{d}'_{79} =$ [	79	2	2	6	2	6	2	10	6	2	10	6	1	14	10	0	1	0	10	14	]$^\mathsf{T}$
Rn	$\mathbf{d}'_{86} =$ [	86	2	2	6	2	6	2	10	6	2	10	6	2	14	10	6	2	6	10	14	]$^\mathsf{T}$

SUPPLEMENTARY TABLE 3: Examples of element descriptors. Here, unscaled descriptors $\mathbf{d}'_Z$ are shown. The entries encode information about the ground state electron configuration (e.g. $1s^2 2s^2 2p^2$ for C), the total number of electrons/nuclear charge (e.g. $Z = 7$ for N), and the number of electrons in the valence shells (e.g. $vs^2 vp^4$ for O). The descriptors used in Eq. 9 in the main text are given by $\mathbf{d}_Z = \mathbf{d}'_Z \oslash \mathbf{d}'_{86}$, where $\oslash$ denotes Hadamard (element-wise) division (such that all entries of $\mathbf{d}_Z$ lie between 0 and 1, which is desirable for numerical reasons). In this work, it is assumed that $Z_{\max} = 86$ covers most practical applications, but descriptors for heavier elements could be derived analogously (and the scaling procedure adapted accordingly if necessary).

loss weights (see Eq. 28 in the main text)		$\alpha_E = \alpha_F = \alpha_\mu = 1$			$\alpha_E = \alpha_\mu = 1$, $\alpha_F = 100$		
		**energy**	**forces**	**dipole**	**energy**	**forces**	**dipole**
**known molecules/ unknown conformations**	MAE	18.513	39.863	39.380	10.620	14.851	121.38
	RMSE	29.885	65.810	58.874	16.782	25.330	165.82
**unknown molecules/ unknown conformations**	MAE	20.490	46.567	41.766	13.151	17.326	120.50
	RMSE	28.740	74.700	61.062	17.891	26.179	162.32

SUPPLEMENTARY TABLE 4: Mean absolute errors (MAEs) and root mean square errors (RMSEs) of energies (meV), forces (meV Å^{-1}) and dipole moments (mD) for the QM7-X[19] dataset. Here, SpookyNet is trained with either a low or high force weight α_F in the loss function (see Eq. 28 in the main text).

Method	MARE	R^2	Spearman ρ
SpookyNet	0.854	0.664	0.612
PaiNN	0.889	0.505	0.430
SchNet	2.565	-0.031	-0.018
GFN2*	0.389	0.637	0.717
B97-3c*	0.198	0.902	0.903
GAFF*	1.638	0.348	0.479

SUPPLEMENTARY TABLE 5: Statistics of the conformer benchmark for different models. Columns indicate median absolute relative error (MARE) in kcal/mol, median R^2 correlation, and median Spearman correlation. The results for the methods marked with an asterisk (*) are taken from Ref. 20 and are measured in relation to DLPNO-CCSD(T) reference data. As such, they are not directly comparable to the values reported for SpookyNet, PaiNN, and SchNet and only listed to give the reader an intuition for the typical range of values.

SUPPLEMENTARY REFERENCES

[1] S. N. Pozdnyakov, M. J. Willatt, A. P. Bartók, C. Ortner, G. Csányi, and M. Ceriotti, Incompleteness of atomic structure representations, Physical Review Letters **125**, 166001 (2020).

[2] A. Glielmo, C. Zeni, and A. De Vita, Efficient nonparametric n-body force fields from machine learning, Physical Review B **97**, 184307 (2018).

[3] O. A. Von Lilienfeld, R. Ramakrishnan, M. Rupp, and A. Knoll, Fourier series of atomic radial distribution functions: A molecular fingerprint for machine learning models of quantum chemical properties, International Journal of Quantum Chemistry **115**, 1084 (2015).

[4] E. Kocer, J. K. Mason, and H. Erturk, Continuous and optimally complete description of chemical environments using spherical Bessel descriptors, AIP Advances **10**, 015021 (2020).

[5] O. T. Unke and M. Meuwly, PhysNet: A neural network for predicting energies, forces, dipole moments, and partial charges, Journal of Chemical Theory and Computation **15**, 3678 (2019).

[6] J. Klicpera, J. Groß, and S. Günnemann, Directional message passing for molecular graphs, arXiv preprint arXiv:2003.03123 (2020).

[7] K. T. Schütt, O. T. Unke, and M. Gastegger, Equivariant message passing for the prediction of tensorial properties and molecular spectra, arXiv preprint arXiv:2102.03150 (2021).

[8] S. Batzner, T. E. Smidt, L. Sun, J. P. Mailoa, M. Kornbluth, N. Molinari, and B. Kozinsky, SE(3)-equivariant graph neural networks for data-efficient and accurate interatomic potentials, arXiv preprint arXiv:2101.03164 (2021).

[9] K. Schütt, P. Kessel, M. Gastegger, K. Nicoli, A. Tkatchenko, and K.-R. Müller, Schnetpack: A deep learning toolbox for atomistic systems, Journal of Chemical Theory and Computation **15**, 448 (2018).

[10] F. A. Faber, A. S. Christensen, B. Huang, and O. A. Von Lilienfeld, Alchemical and structural distribution based representation for universal quantum machine learning, The Journal of Chemical Physics **148**, 241717 (2018).

[11] A. S. Christensen, L. A. Bratholm, F. A. Faber, and O. Anatole von Lilienfeld, Fchl revisited: Faster and more accurate quantum machine learning, The Journal of Chemical Physics **152**, 044107 (2020).

[12] A. S. Christensen, F. A. Faber, B. Huang, L. A. Bratholm, A. Tkatchenko, K. R. Müller, and O. A. von Lilienfeld, QML: A python toolkit for quantum machine learning, `https://www.qmlcode.org` (2017).

[13] J. Behler and M. Parrinello, Generalized neural-network representation of high-dimensional potential-energy surfaces, Physical Review Letters **98**, 146401 (2007).

[14] K. T. Schütt, H. E. Sauceda, P.-J. Kindermans, A. Tkatchenko, and K.-R. Müller, SchNet – a deep learning architecture for molecules and materials, The Journal of Chemical Physics **148**, 241722 (2018).

[15] S. Pozdnyakov, M. Willatt, and M. Ceriotti, Randomly-displaced methane configurations, Materials Cloud Archive 2020.110 (2020), `https://doi.org/10.24435/materialscloud:qy-dp`.

[16] B. Huang and O. A. Von Lilienfeld, Communication: Understanding molecular representations in machine learning: The role of uniqueness and target similarity, The Journal of Chemical Physics **145**, 161102 (2016).

[17] F. L. Hirshfeld, Bonded-atom fragments for describing molecular charge densities, Theoretica Chimica Acta **44**, 129 (1977).

[18] J. Hoja, L. M. Sandonas, B. Ernst, A. Vazquez-Mayagoitia, R. A. J. DiStasio, and A. Tkatchenko, QM7-X: A comprehensive dataset of quantum-mechanical properties spanning the chemical space of small organic molecules, (Version 1.0) [Data set] Zenodo (2020), `http://doi.org/10.5281/zenodo.3905361`.

[19] J. Hoja, L. M. Sandonas, B. G. Ernst, A. Vazquez-Mayagoitia, R. A. DiStasio Jr, and A. Tkatchenko, QM7-X, a comprehensive dataset of quantum-mechanical properties spanning the chemical space of small organic molecules, Scientific Data **8**, 43 (2021).

[20] D. Folmsbee and G. Hutchison, Assessing conformer energies using electronic structure and machine learning methods, International Journal of Quantum Chemistry **121**, e26381 (2021).
